# Seizure-Related Deterioration in Treated Glioma: A Narrative Review of Nonconvulsive Status Epilepticus, Peri-Ictal Pseudoprogression, Treatment-Related Change, and True Progression

**DOI:** 10.3390/diagnostics16162572

**Published:** 2026-08-14

**Authors:** Polina Chliapnikov, Mark Bernstein

**Affiliations:** Division of Neurosurgery, Toronto Western Hospital, University of Toronto, 399 Bathurst St., Toronto, ON M5T 2S8, Canada; mark.bernstein@uhn.ca

**Keywords:** glioma, nonconvulsive status epilepticus, peri-ictal pseudoprogression, tumor-related epilepsy, classic pseudoprogression, radiation necrosis, true progression

## Abstract

**Background/Objectives:** In glioma patients, acute neurologic deterioration may reflect nonconvulsive status epilepticus (NCSE), peri-ictal pseudoprogression, treatment-related change, or true progression. As these entities share imaging features, misdiagnosis can delay therapy. This review synthesizes evidence relevant to a proposed electroclinical-imaging framework for distinguishing seizure-related deterioration from treatment-related change and true progression. **Methods:** This narrative review synthesized English-language literature on tumor-related epilepsy, NCSE, peri-ictal imaging abnormalities, pseudoprogression, radiation necrosis, Response Assessment in Neuro-Oncology (RANO) criteria, and advanced imaging in adult diffuse glioma. **Results:** Seizure recurrence after glioma treatment may indicate tumor activity, but it may also reflect radiation injury, postoperative gliosis, edema, metabolic disturbance, medication effects, or transient peri-ictal change. NCSE is particularly important because it may present with aphasia, confusion, impaired consciousness, behavioral change, or deficits without convulsions, making electroencephalography central for unexplained deterioration. Seizure activity can also produce transient magnetic resonance imaging (MRI) or positron emission tomography (PET) abnormalities, including enhancement, fluid-attenuated inversion recovery (FLAIR) hyperintensity, diffusion restriction, and perfusion changes that mimic recurrence. **Conclusions:** This review proposes a structured electroclinical-imaging framework incorporating early EEG, seizure history, treatment timing, corticosteroid and antiseizure response, multimodal imaging, and longitudinal reassessment for prospective evaluation in treated glioma patients with new neurologic decline.

## 1. Introduction

### 1.1. Post-Treatment Glioma and Diagnostic Uncertainty

Adult diffuse gliomas remain among the most clinically complex tumors in neuro-oncology because they are shaped not only by infiltrative biology, but also by treatment-related injury, seizure burden, evolving molecular classification, and the limitations of conventional imaging surveillance. The fifth edition of the World Health Organization classification has reinforced the central role of molecular diagnosis, grouping adult-type diffuse gliomas into glioblastoma, isocitrate dehydrogenase (IDH)-wildtype; astrocytoma, IDH-mutant; and oligodendroglioma, IDH-mutant and 1p/19q-codeleted [1]. These molecular entities should not be treated as clinically interchangeable. IDH-mutant astrocytomas and oligodendrogliomas are generally more seizure-prone and may contain substantial nonenhancing disease, whereas IDH-wildtype glioblastoma commonly follows a more aggressive enhancing course and is frequently evaluated during the early post-chemoradiation interval, when classic pseudoprogression is a major consideration [2]. These differences influence seizure presentation, treatment exposure, expected progression patterns, and the interpretation of post-treatment imaging. Treatment plans are often structured to emphasize maximal safe resection followed by risk-adapted radiotherapy, chemotherapy, and longitudinal magnetic resonance imaging (MRI) assessment [2]. Specifically for glioblastoma, the introduction of radiotherapy with adjuvant temozolomide established the modern chemoradiation approach, improving survival while also increasing the frequency with which clinicians must interpret early and post-treatment imaging abnormalities [3]. With prolonged survival and clinical-imaging surveillance, new neurologic deterioration in treated glioma patients must be interpreted within a multifactorial post-treatment context rather than exclusive attribution to tumor progression. Potential etiologies include true progression, treatment-related injury, corticosteroid modification, vasogenic edema, infection, vascular complications, metabolic derangements, medication toxicity, and seizure-related dysfunction [4]. Seizure-related deterioration is especially challenging because it may overlap with tumor progression in clinical presentation, while remaining potentially reversible with timely recognition and treatment [5].

In this review, these concepts are considered components of a single post-treatment diagnostic problem rather than isolated clinical entities. Glioma provides the underlying disease context, in which infiltrative tumor growth creates a sustained risk of neurologic instability. Tumor-related epilepsy represents one of the most frequent and clinically meaningful manifestations of glioma, but seizure recurrence after treatment may reflect either tumor activity or irritative changes [5]. Nonconvulsive status epilepticus (NCSE) is particularly important because it may produce neurologic deterioration without overt convulsions, making electroencephalographic assessment vital in diagnosis [6]. Peri-ictal pseudoprogression describes seizure-associated changes that can simulate tumor growth, while true progression refers to genuine biologic tumor advancement requiring oncologic intervention [7]. The term peri-ictal refers to the period immediately before, during, and after seizure activity, when transient physiologic changes in cortical excitability, perfusion, edema, and blood–brain barrier permeability may alter imaging appearance [8]. Considering these entities together provides a structure for examining their overlapping clinical and imaging features but does not establish that any individual feature or combination of features can reliably distinguish them.

### 1.2. Seizure-Related Clinical and Imaging Mimics of Progression

Seizures are considered to be one of the defining clinical features of glioma. They may be the presenting symptom of low-grade and IDH-mutant tumors, complicate the course of high-grade gliomas, and re-emerge during recurrence or treatment-related irritation. Reviews of glioma-related epilepsy consistently describe seizures as common, disabling, and clinically informative, with incidence varying by tumor grade, cortical location, molecular profile, extent of resection, and disease phase [5]. Although seizures at initial presentation have been associated in some high-grade gliomas with more favorable tumor biology, the escalation of seizures after treatment is commonly interpreted as a potential marker of tumor recurrence [9]. However, seizure recurrence is not specific for tumor growth and requires concurrent clinical, EEG, imaging, and longitudinal assessment [5,7].

This diagnostic uncertainty is most clinically significant when seizure activity is nonconvulsive, as NCSE may present with confusion, aphasia, abulia, fluctuating focal deficits, reduced consciousness, or subtle behavioral change rather than overt convulsions [6]. In treated glioma patients, these manifestations may be difficult to distinguish from tumor progression, radiation necrosis, corticosteroid-responsive edema, medication toxicity, or metabolic encephalopathy [10]. For this reason, electroencephalography (EEG)—a non-invasive test that records cortical electrical activity to detect abnormal brain activity patterns—is central when neurologic decline is unexplained, fluctuating, or disproportionate to imaging findings [11]. EEG interpretation in neuro-oncology remains complex because glioma patients may have structural lesions, postoperative changes, periodic discharges, and abnormal baseline cortical activity even in the absence of ongoing seizures [11,12].

Peri-ictal abnormalities reflect overlapping processes, including altered tissue water content, impaired water diffusion, blood–brain barrier disruption, and regional perfusion change [8]. In treated glioma, these abnormalities may arise within or near the tumor bed, where residual infiltrating tumor, radiation injury, postoperative change, edema, and epileptogenic cortex may already coexist. Peri-ictal pseudoprogression captures this overlap, describing seizure-associated clinical and imaging changes that can simulate tumor growth [7]. Simultaneously, glioma patients are also vulnerable to non-seizure treatment effects, including pseudoprogression and radiation necrosis, which can also produce enhancement, edema, neurologic decline, and corticosteroid responsiveness [13]. Pseudoprogression refers to treatment-related imaging worsening that subsequently stabilizes or improves without representing true tumor growth, whereas radiation necrosis reflects delayed radiation-associated tissue injury that may produce necrosis, edema, enhancement, and mass effect [13]. Although conventional MRI, perfusion and diffusion imaging, MRS, PET, and Response Assessment in Neuro-Oncology (RANO) criteria improve post-treatment evaluation, none fully resolves the confounding effect of seizure-related deterioration in adult-type diffuse glioma patients [14,15]. RANO criteria provide standardized rules for assessing glioma response and progression by integrating imaging changes with clinical status and corticosteroid use, but they do not specifically function as a diagnostic algorithm for seizure-related neurologic deterioration [16]. This limitation reflects the broader evolution of glioma response assessment, from enhancement-centered Macdonald criteria to RANO-based frameworks that better account for non-enhancing disease, corticosteroid exposure, clinical status, and treatment-related imaging change [16,17,18,19].

### 1.3. Rationale and Aims of This Review

Although tumor-related epilepsy, status epilepticus, peri-ictal imaging abnormalities, pseudoprogression, radiation necrosis, and tumor progression are all considered in routine neuro-oncology practice, the evidence relevant to their differentiation is distributed across several distinct bodies of literature. Glioma epilepsy studies often focus on seizure mechanisms and antiseizure management [5]. NCSE studies emphasize electrographic diagnosis [11]. The pseudoprogression literature generally centers on response assessment, while imaging studies focus on distinguishing treatment effect from tumor recurrence [13,14]. This review therefore brings these bodies of evidence together for treated glioma patients who present with acute or subacute neurologic deterioration, in whom NCSE, peri-ictal pseudoprogression, treatment-related change, and true progression may all remain plausible.

The consequences of misclassification are clinically significant. Missed NCSE may delay urgent antiseizure treatment and allow potentially reversible neurologic dysfunction to be mislabeled as irreversible progression [6,10]. Conversely, misinterpreting peri-ictal imaging change as recurrence may lead to unnecessary oncologic escalation, biopsy, open surgery, treatment discontinuation, or inaccurate counselling [7]. Attributing true progression to seizure-related or treatment-related change may delay appropriate therapy [16]. Although these elements are already incorporated into routine assessment, a structured synthesis may help clarify how seizure timing, EEG findings, treatment interval, corticosteroid and antiseizure medication response, multimodal imaging, and longitudinal follow-up should be interpreted when several diagnoses remain plausible [14,16].

Accordingly, the research question guiding this narrative review is: in treated glioma patients who develop acute or subacute neurologic deterioration, what features can help distinguish NCSE, peri-ictal pseudoprogression, treatment-related change, and true tumor progression? The aims are to (1) summarize the clinical presentations and mechanisms by which seizures and NCSE produce neurologic decline in treated glioma patients; (2) review peri-ictal MRI and metabolic imaging changes that may mimic progression; (3) compare these findings with established patterns of pseudoprogression, radiation injury, and true progression; and (4) present a proposed clinically oriented framework that organizes early EEG, careful seizure history, antiseizure treatment response, imaging findings, treatment context, and longitudinal follow-up. This review does not assume that these conditions are routinely considered in isolation; rather, it synthesizes the evidence supporting their differentiation and identifies the limitations that require prospective validation.

## 2. Materials and Methods

This article was designed as a narrative review focused on seizure-related neurologic deterioration in treated glioma patients, with particular emphasis on the diagnostic overlap among nonconvulsive status epilepticus (NCSE), peri-ictal pseudoprogression, treatment-related change, and true tumor progression. Relevant sources were selected purposively according to their direct relevance to the review question. The objective was to synthesize the clinical, EEG-based, and imaging literature relevant to this diagnostic problem and to identify features that may help distinguish reversible seizure-related deterioration from progressive disease in patients with previously treated diffuse glioma. The reporting approach was informed by principles for the transparent presentation of narrative reviews [17].

### Search Strategy and Evidence Synthesis

A literature search was conducted using PubMed/MEDLINE, EMBASE, Scopus, and Web of Science. The search was intended to identify representative and clinically relevant literature for a narrative synthesis rather than to serve as an exhaustive systematic search. Searches were limited to English-language peer-reviewed sources and included articles published up to April 28, 2026. Search terms were selected to capture four overlapping domains: glioma and brain tumor-related epilepsy; NCSE and electroencephalographic diagnosis; peri-ictal and post-ictal imaging abnormalities; and post-treatment response assessment in glioma. Search terms included combinations of “glioma,” “glioblastoma,” “diffuse glioma,” “treated glioma,” “brain tumor-related epilepsy,” “tumor-related epilepsy,” “seizure,” “status epilepticus,” “NCSE,” “electroencephalography,” “EEG,” “peri-ictal MRI,” “postictal MRI,” “peri-ictal pseudoprogression,” “pseudoprogression,” “radiation necrosis,” “treatment effect,” “true progression,” “tumor recurrence,” “RANO,” “perfusion MRI,” “diffusion-weighted imaging,” “MR spectroscopy,” “PET,” and “amino acid PET.” Search terms were combined iteratively within each database; because the search was purposive and narrative, no single fixed database-specific Boolean strategy was applied.

Sources were considered relevant if they addressed at least one of the following areas: seizures or status epilepticus in glioma or brain tumor populations; clinical or electroencephalographic diagnosis of NCSE; seizure-associated MRI or PET abnormalities; peri-ictal pseudoprogression in brain tumor or glioma patients; post-treatment imaging change after glioma therapy; radiation necrosis; pseudoprogression; true progression; or response-assessment criteria used to distinguish treatment effect from tumor recurrence. The narrative synthesis considered prospective and retrospective clinical studies, cohort studies, case series, case reports, systematic reviews, narrative reviews, clinical guidelines, and imaging response-assessment frameworks. Broader brain tumor, epilepsy, neuroimaging, or critical-care EEG studies were included when they provided directly relevant information on NCSE diagnosis, peri-ictal imaging findings, or post-treatment response assessment. Articles were excluded if they focused exclusively on pediatric tumors, non-glioma neurologic diseases, primary epilepsy syndromes, or imaging abnormalities unrelated to seizure activity, treatment effect, or glioma progression.

Evidence synthesis was organized around diagnostic domain and clinical utility. Findings were organized according to clinical features, EEG features, conventional MRI findings, advanced imaging findings, treatment response, longitudinal follow-up, and consequences of misclassification. Particular attention was given to features that may help differentiate seizure-driven deterioration from progression, including fluctuating deficits, altered consciousness, aphasia, timing relative to seizure activity, EEG-confirmed electrographic seizures, transient cortical or subcortical T2/FLAIR hyperintensity, diffusion restriction, perfusion changes, enhancement patterns, corticosteroid responsiveness, antiseizure medication response, and imaging evolution on follow-up imaging. Because the evidence base was heterogeneous, the design and principal limitations of the key studies are identified in the narrative synthesis. No formal risk-of-bias tool or evidence-grading system was applied because this review was designed as a narrative synthesis.

## 3. Results

### 3.1. Post-Treatment Glioma as a Framework for Interpreting Neurologic Deterioration

The core diagnostic problem arises from the biology and treatment course of adult diffuse gliomas. These tumors are infiltrative, spatially and histologically heterogeneous, and frequently located adjacent to functional cortex, creating a risk of focal neurologic dysfunction and epileptogenesis—the development of epilepsy [1,5]. The European Association of Neuro-Oncology (EANO) guidelines emphasize that adult diffuse gliomas require integrated diagnostic assessment and management, typically involving maximal safe resection, radiotherapy, systemic therapy, and longitudinal imaging surveillance [2]. In the post-treatment setting, neurologic deterioration is rarely attributable to one isolated mechanism. Tumor progression remains a major concern, but the treated brain may also show radiation-induced inflammation, pseudoprogression, radiation necrosis, vasogenic edema, corticosteroid-related fluctuation, vascular complications, infection, metabolic disturbance, medication toxicity, and seizure-related dysfunction—some of which may coexist [2,13]. A patient with residual glioma may develop peri-lesional edema after steroid taper, experience nonconvulsive seizures from cortical irritation, and show enhancement that reflects either treatment effect or tumor recurrence [10,13]. The clinical presentation may be focal neurological deficit, cognitive slowing, altered consciousness, or functional decline. However, none of these findings alone reliably distinguish seizure-related deterioration from progressive disease [5,10,13].

Conventional response assessment has improved but has not eliminated this ambiguity. Before RANO, the Macdonald criteria provided the dominant imaging response framework for supratentorial malignant glioma, relying primarily on bidimensional contrast-enhancing tumor measurements while also considering corticosteroid use and neurologic status [18]. The original RANO criteria were developed to address limitations of older contrast-enhancement-based response systems by incorporating corticosteroid use and clinical status [16]. RANO 2.0 further standardized response assessment across gliomas and emphasized the need for confirmatory imaging when pseudoprogression is likely [19]. However, RANO was not designed to function as an acute seizure-differential framework; in routine practice, its response-assessment criteria must be interpreted alongside neurologic symptoms, seizure history, EEG findings, corticosteroid and antiseizure medication exposure, and interval MRI or PET evolution [7,8,10,16,19]. This additional interpretation reflects established multidisciplinary assessment rather than a fundamentally new diagnostic approach.

### 3.2. Tumor-Related Epilepsy as Both a Symptom and Confounder of Progression

Tumor-related epilepsy is a common and clinically meaningful manifestation of glioma [5,9]. Seizures may occur at diagnosis, after resection, during radiotherapy or chemotherapy, at recurrence, or near end of life [5]. Their frequency varies by tumor type, cortical location, grade, molecular profile, and treatment phase [5,9]. Current reviews emphasize that tumor-related epilepsy affects quality of life, functional independence, driving, employment, neurocognitive function, psychological health, and treatment decision-making [5,9]. Therefore, seizure control is part of the clinical phenotype of glioma rather than merely a supportive-care outcome [5]. Low-grade and IDH-mutant gliomas are especially epileptogenic, partly because they infiltrate cortical networks over longer periods and may alter neuronal excitability without immediately destroying the surrounding cortex [9,20]. IDH-mutant gliomas produce D-2-hydroxyglutarate, which has been experimentally linked to epileptogenesis through metabolic effects on peritumoral neurons [20]. Gliomas may also promote seizures through glutamate release, impaired uptake, blood–brain barrier disruption, inflammatory changes, peritumoral hypoxia, and aberrant tumor–neuronal interactions [21,22]. These mechanisms explain why seizures can arise from active bidirectional interactions between tumor cells, peritumoral cortex, and neural circuits [21,22]. The clinical significance of seizure recurrence may therefore differ across glioma subtypes, although many available studies combine tumor classes or predate contemporary molecular classification [5,9].

Clinically, seizures may have different implications depending on timing [9]. Seizures at initial presentation—especially in IDH-mutant or lower-grade gliomas—may correlate with a less aggressive course because they bring attention to tumors earlier [9]. In contrast, new or worsening seizures after initial therapy often raise clinical concern for recurrence [5,9]. Recent studies support an association between seizure control and tumor status, including the development of RANO-TREAT—a standardized seizure assessment tool intended for glioma trials and clinical practice [23]. RANO-TREAT acknowledges that seizure outcomes should be measured systematically alongside MRI and traditional oncologic endpoints [23]. This builds on earlier work proposing seizure control as a clinically meaningful metric in low-grade glioma treatment trials [24]. It is further supported by recent evidence that seizure-related endpoints remain substantially underreported in adult diffuse glioma trials [25]. A recent review of ClinicalTrials.gov found that only 65 of 2801 adult-type diffuse glioma trials initiated since 2010 included seizure-related outcomes, highlighting the persistent underrepresentation and heterogeneity of seizure endpoints in glioma research [25]. However, the association between seizure burden and progression is not specific enough to treat seizure recurrence as equivalent to tumor growth [5,7].

Worsening seizures may accompany progressive tumor, but they may also result from postoperative gliosis, radiotherapy-related cortical injury, radiation necrosis, perilesional edema, missed antiseizure medication doses, subtherapeutic exposure, drug interactions, infection, electrolyte disturbance, sleep deprivation, or transient peri-ictal changes [5,7]. Conversely, a seizure may produce neurologic deficits and imaging abnormalities that mimic progression even when the tumor is stable [7,8]. This creates diagnostic risk, as seizures may be interpreted as evidence of progression, while seizure-induced MRI abnormalities reinforce that interpretation [7,8]. The literature therefore supports seizure recurrence as a warning signal requiring reassessment, not as a surrogate for progression [5,7].

### 3.3. NCSE as a Reversible Mimic of Glioma Progression

Diagnostic problems are most urgent when seizures are nonconvulsive. Status epilepticus is defined by persistent seizure activity or recurrent seizures without return to baseline, with the International League Against Epilepsy (ILAE) framework emphasizing time-dependent thresholds for treatment urgency and risk of neuronal injury [26]. Aphasia, confusion, decreased responsiveness, impaired attention, fluctuating hemiparesis, visual symptoms, behavioral change, or unexplained decline may be interpreted as tumor progression, radiation necrosis, edema, metabolic encephalopathy, or medication toxicity [6,10]. Without EEG, ongoing electrographic seizure activity may remain undetected, making EEG central to the evaluation of unexplained neurologic decline [6,10]. The Salzburg criteria were developed to provide a structured approach to diagnosing NCSE on EEG [11]. The American Clinical Neurophysiology Society (ACNS) standardized critical-care EEG terminology and provides a common vocabulary for electrographic seizures, electrographic status epilepticus, periodic discharges, and rhythmic activity patterns [12]. However, EEG interpretation in glioma patients remains challenging because structural lesions, postoperative cavities, cortical injury, and focal slowing may produce abnormal EEG backgrounds even without ongoing seizures [10,12]. In this setting, the diagnostic question is whether the pattern explains the clinical syndrome and improves with antiseizure treatment [10,12].

In a retrospective glioma cohort, Knudsen-Baas et al. found that status epilepticus secondary to glioma often responded to treatment and should be managed actively regardless of oncologic prognosis [27]. In a retrospective observational study, Giovannini et al. described the clinical characteristics and outcomes of tumor-associated status epilepticus in adults with glioma [28]. Stritzelberger et al. reported that nearly 10% of patients with glioblastoma in a single-center cohort experienced status epilepticus, and that status epilepticus was successfully treated in many cases despite poor oncologic prognosis [29]. Rickel et al.’s multicenter cohort study of primary brain tumors and metastases similarly found that status epilepticus was associated with functional decline across tumor types [30]. Together, these studies support the clinical relevance and potential treatability of status epilepticus in neuro-oncology, although their observational designs and heterogeneous populations limit subtype-specific conclusions [27,28,29,30]. Clinically, treated glioma patients with acute neurologic decline should not be assumed to have irreversible tumor progression before electrographic seizure activity is excluded [6,10]. Specifically, aphasia may suggest dominant temporal or frontal progression; hemiparesis may suggest expanding edema or corticospinal tract involvement; confusion may suggest multifocal progression or medication toxicity [6,10]. The evidence supports early EEG when deterioration is unexplained by imaging or accompanied by subtle symptoms [6,10,11]. Routine EEG may be used initially in clinically stable patients with unexplained focal or fluctuating symptoms. Continuous EEG should be considered when impaired consciousness or fluctuating deficits persist, events recur, or clinical suspicion remains high despite a nondiagnostic routine recording [6,10,12].

### 3.4. Peri-Ictal Imaging Abnormalities and Imaging Mimicry

Seizure-related deterioration becomes misleading when neurologic decline is accompanied by MRI abnormalities [7,8]. Seizures can produce transient changes on MRI, including cortical or subcortical T2/FLAIR hyperintensity, gyral swelling, diffusion restriction, reduced ADC, contrast enhancement, and perfusion abnormalities [8]. A comprehensive review by Williams et al. of peri-ictal MRI changes identified hundreds of reported cases and found that seizure-induced imaging abnormalities most commonly involved diffusion restriction and reduced ADC in cortical and mesial temporal regions [8]. In patients without tumors, peri-ictal MRI changes may be mistaken for stroke, encephalitis, demyelination, or neoplasm [8]. In treated glioma, the risk of misinterpretation is increased because the seizure focus often overlaps anatomically with the tumor bed [7]. A region of postoperative change, residual non-enhancing tumor, radiation injury, and epileptogenic cortex may develop transient enhancement or FLAIR abnormality after seizures [7,8]. Without attention to seizure timing, these findings may be misclassified as recurrence [7]. Peri-ictal hyperperfusion may complicate MRI interpretation, diffusion restriction may be mistaken for hypercellular tumor, and metabolic changes may confound PET or spectroscopy, illustrating how seizure-related imaging change can imitate features typically used to infer viable tumor [7,8,31].

Rheims et al. described transient seizure-related MRI abnormalities in brain tumor patients that wrongly suggested tumor progression, helping define peri-ictal pseudoprogression as a distinct post-treatment diagnostic pitfall [7]. The subsequent literature has shown that post-ictal changes may present as late pseudoprogression on MRI and PET in diffuse glioma, further emphasizing that seizure-related changes can occur outside the early pseudoprogression window [31]. Several features may support peri-ictal pseudoprogression, although none are definitive alone [7]. Suggestive features include close temporal association with seizures or NCSE; cortical or gyriform enhancement; FLAIR or diffusion abnormalities following cortical networks rather than a single vascular territory; hyperperfusion in an ictal region; discordance between substantial symptoms and limited mass effect; improvement after antiseizure treatment; and partial or complete reversal of diffusion and other imaging abnormalities on follow-up [7,8,31]. However, glioma progression can also provoke seizures and may itself show cortical or infiltrative patterns [5,9]. Therefore, peri-ictal pseudoprogression should be treated as a diagnostic possibility requiring active testing rather than as a default explanation for all post-seizure enhancement [7,8,31].

The literature also suggests that delayed seizure-related imaging phenomena may overlap with other post-radiation syndromes [32]. Stroke-like migraine attacks after radiation therapy (SMART) syndrome and related radiation-induced cortical phenomena can present with seizures, migraine-like episodes, focal deficits, and transient cortical enhancement years after cranial irradiation [32]. Although not identical to peri-ictal pseudoprogression, these syndromes reinforce the broader principle that prior radiotherapy can create delayed cortical vulnerability, making seizure-associated enhancement and neurologic deficits possible long after initial treatment [32]. In treated glioma surveillance, timing alone therefore cannot fully exclude seizure-related or treatment-related mimics [7,32].

### 3.5. Pseudoprogression, Radiation Necrosis, and Treatment-Related Change

Treatment-related imaging change is already one of the most established mimics of glioma progression [13]. Pseudoprogression classically refers to new or increased enhancement and/or edema after therapy that later stabilizes without an increase in tumor burden [13]. It is particularly recognized after radiotherapy with concomitant temozolomide in glioblastoma, often within the first 12 weeks after chemoradiation, although later presentations may occur [13,33]. Taal et al. reported that up to 50% of malignant glioma patients with apparent progression immediately after radiotherapy and temozolomide developed pseudoprogression rather than true early progression [33]. Brandes et al. showed that O6-methylguanine-DNA methyltransferase (MGMT) promoter methylation was associated with the incidence and outcome of pseudoprogression after concomitant radiochemotherapy in newly diagnosed glioblastoma [34]. Radiation necrosis is generally considered a later and more destructive form of treatment-related injury, often associated with vascular damage, inflammation, necrosis, edema, mass effect, and corticosteroid responsiveness. Both pseudoprogression and radiation necrosis may cause clinical deterioration and imaging worsening, making them difficult to distinguish from viable tumor. Thust, Van den Bent and Smits emphasized that pseudoprogression is not only an imaging phenomenon but also a major clinical and trial-level problem, because it can interfere with routine patient management, response assessment, and interpretation of therapeutic efficacy [13].

The biological basis of treatment-related change includes endothelial injury, increased vascular permeability, inflammatory cell infiltration, demyelination, tissue necrosis, and disruption of the blood–brain barrier [13]. These processes can produce contrast enhancement and vasogenic edema on MRI, the same findings that often trigger concern for progression [13]. MGMT promoter methylation has been associated with pseudoprogression after chemoradiation, likely reflecting greater treatment sensitivity and tumor-cell injury in methylated glioblastoma [34]. However, molecular and clinical predictors do not provide definitive diagnosis in individual patients, as a patient with unmethylated MGMT can still develop treatment effect, and a patient with methylated MGMT can still progress early [13,34]. RANO and RANO 2.0 directly address this challenge by recommending caution in diagnosing early progression after radiotherapy and by emphasizing confirmatory imaging in settings where pseudoprogression is plausible [16,19]. These criteria are essential for trial consistency and clinical communication, but they do not fully resolve acute deterioration because clinical status and corticosteroid use can be influenced by seizures [16,19]. If the patient improves after antiseizure therapy and imaging improves on short-interval follow-up, the original episode may represent seizure-related deterioration or peri-ictal pseudoprogression [7,8]. The evidence therefore supports conceptual separation between classic treatment-related change, seizure-related imaging change, and true progression [7,13,16,19]. Overall, classic pseudoprogression is usually framed around therapy-induced inflammatory or vascular effects; peri-ictal pseudoprogression is framed around seizure-associated transient imaging abnormality; and true progression reflects biologic tumor growth [16,19]. In practice, these categories can overlap [7,13,16,19]. The clinician’s task is not always to assign one label, but to determine which mechanism is dominant enough to guide management [7,13,16,19].

### 3.6. True Progression and Imaging Limitations

True progression remains the most consequential alternative diagnosis because delayed oncologic treatment can worsen outcome [16,19]. Progression may present with increasing enhancement, expanding non-enhancing FLAIR abnormality, rising perfusion, new diffusion abnormalities, increased amino-acid uptake, mass effect, steroid dependence, worsening deficits, and seizure recurrence [14,16,19]. The relative importance of these findings differs by molecular class: progression assessment in IDH-wildtype glioblastoma primarily emphasizes enhancing disease, whereas both enhancing and nonenhancing components may remain relevant in IDH-mutant astrocytoma and oligodendroglioma [19]. Conventional MRI reflects blood–brain barrier disruption rather than tumor burden itself [13,16]. Non-enhancing FLAIR abnormality can represent infiltrative tumor but also edema, gliosis, seizure-related change, ischemia, demyelination, or treatment effect [13,16,19]. Clinical decline can support progression but may be confounded by NCSE, steroid taper, infection, medication toxicity, or metabolic disturbance [10,13,16]. Histopathology is often treated as a reference standard, but even tissue diagnosis may be limited by sampling error and mixed pathology [13,16]. Surgical specimens from suspicious enhancing lesions may contain variable proportions of recurrent tumor, necrosis, reactive gliosis, macrophage infiltration, treatment effect, and viable infiltrating cells [13,16]. Therefore, true progression is best established through convergent evidence: serial growth on imaging, progressive clinical decline despite correction of reversible factors, metabolic or perfusion evidence of viable tumor, histologic confirmation when available, and lack of resolution after seizure control or steroid optimization [13,16,19].

The current literature increasingly supports standardized, multimodal interpretation rather than reliance on conventional MRI alone [14,15,16,19]. RANO 2.0 is important because it updates response assessment for adult gliomas and provides a more unified framework for high- and low-grade glioma trials, but does not replace clinical judgment when seizure-related confounders are present [19]. The evidence therefore supports a staged diagnostic approach: stabilize and assess for seizures early, interpret MRI in treatment and seizure context, and use advanced imaging or clinically timed repeat MRI when immediate classification remains uncertain [7,8,14,16,19]. The development of RANO-TREAT is especially relevant because it reflects a growing recognition that seizure burden should be measured systematically in glioma care and trials [23]. Historically, seizure outcomes have been inconsistently captured in glioma studies despite their effect on function and quality of life [24,25]. The recent literature describes seizure outcomes as an understudied metric in glioma clinical trials and emphasizes the need for standardized seizure definitions and longitudinal tracking [25]. Seizure worsening may be both a clinical endpoint and a diagnostic confounder, and without standardized seizure assessment, clinicians risk conflating tumor response, seizure control, and treatment toxicity [23,24,25].

### 3.7. Advanced Imaging: Value, Limitations, and Seizure Confounding

Advanced imaging techniques can improve the distinction between treatment effect and recurrent tumor but do not eliminate uncertainty [14,15]. Perfusion MRI is widely used because recurrent tumor often demonstrates increased vascularity and relative cerebral blood volume, whereas treatment effect may show lower perfusion [14]. Diffusion-weighted imaging may identify hypercellular tumor through restricted diffusion, although diffusion restriction can also occur in ischemia, seizure-related cytotoxic edema, abscess, and treatment injury [8,14]. MRS may provide metabolic information, including choline elevation, N-acetylaspartate reduction, lactate, and lipid peaks, but spatial heterogeneity and overlap with necrosis limit specificity [14]. Radiomics and machine-learning approaches remain investigational because they require standardized acquisition, external validation, and evidence of clinical utility before routine use [35].

PET imaging has become an important adjunct, particularly amino-acid PET using radioactively labeled amino acid tracers (such as O-(2-[18F]fluoroethyl)-L-tyrosine (FET), [11C] methionine (MET), and 6-[18F] fluoro-L-3,4-dihydroxyphenylalanine (FDOPA)) [15]. Joint guidelines from the European Association of Nuclear Medicine (EANM), EANO, RANO group, and Society of Nuclear Medicine and Molecular Imaging (SNMMI) support standardized PET acquisition and interpretation in glioma, and amino-acid PET is increasingly recognized as useful for differentiating tumor recurrence from treatment-related changes [15]. PET RANO 1.0 subsequently proposed standardized PET-based response-assessment criteria for diffuse glioma trials, although these criteria remain primarily trial-oriented and require further prospective validation. The 2025 RANO/EANO PET update further strengthens this position by providing contemporary recommendations for the clinical use of PET imaging in glioma, reflecting the growing role of amino-acid PET in diagnostic clarification, treatment planning, and post-treatment assessment [36]. Both PET and perfusion-weighted imaging have diagnostic value for differentiating recurrence or progression from radiation necrosis in post-treatment gliomas, but performance varies across modalities [14]. Peri-ictal hyperperfusion may mimic tumor-related neovascularity on perfusion MRI, while seizure-related diffusion restriction may mimic hypercellularity [8]. Post-ictal metabolic changes may alter fluorodeoxyglucose (FDG) uptake or amino acid tracer interpretation [31]. Nair et al.’s case report is particularly relevant because it described post-ictal changes presenting as late pseudoprogression on both MRI and PET in a diffuse glioma patient, demonstrating that even metabolic imaging may be confounded by recent seizure activity [31]. The most reliable interpretation often comes from serial multimodal assessment rather than a single time point [13,16,19]. In uncertain cases, short-interval MRI after seizure stabilization may be more informative than immediate oncologic escalation [7,8,16,19].

### 3.8. Diagnostic Integration

The available evidence can be organized within a proposed framework built around four domains: clinical pattern, electroencephalographic confirmation, imaging phenotype, and longitudinal evolution [6,7,8,9,10,13,16,19]. Seizure-related deterioration should be suspected when neurologic deficits are abrupt, fluctuating, episodic, or disproportionate to imaging burden, particularly when language, awareness, behavior, or focal motor function is affected [5,6,10]. However, clinical presentation alone is insufficient because true progression may also present subacutely, and seizures may occur as a manifestation of recurrence [5,9,16].

**Figure 1:**
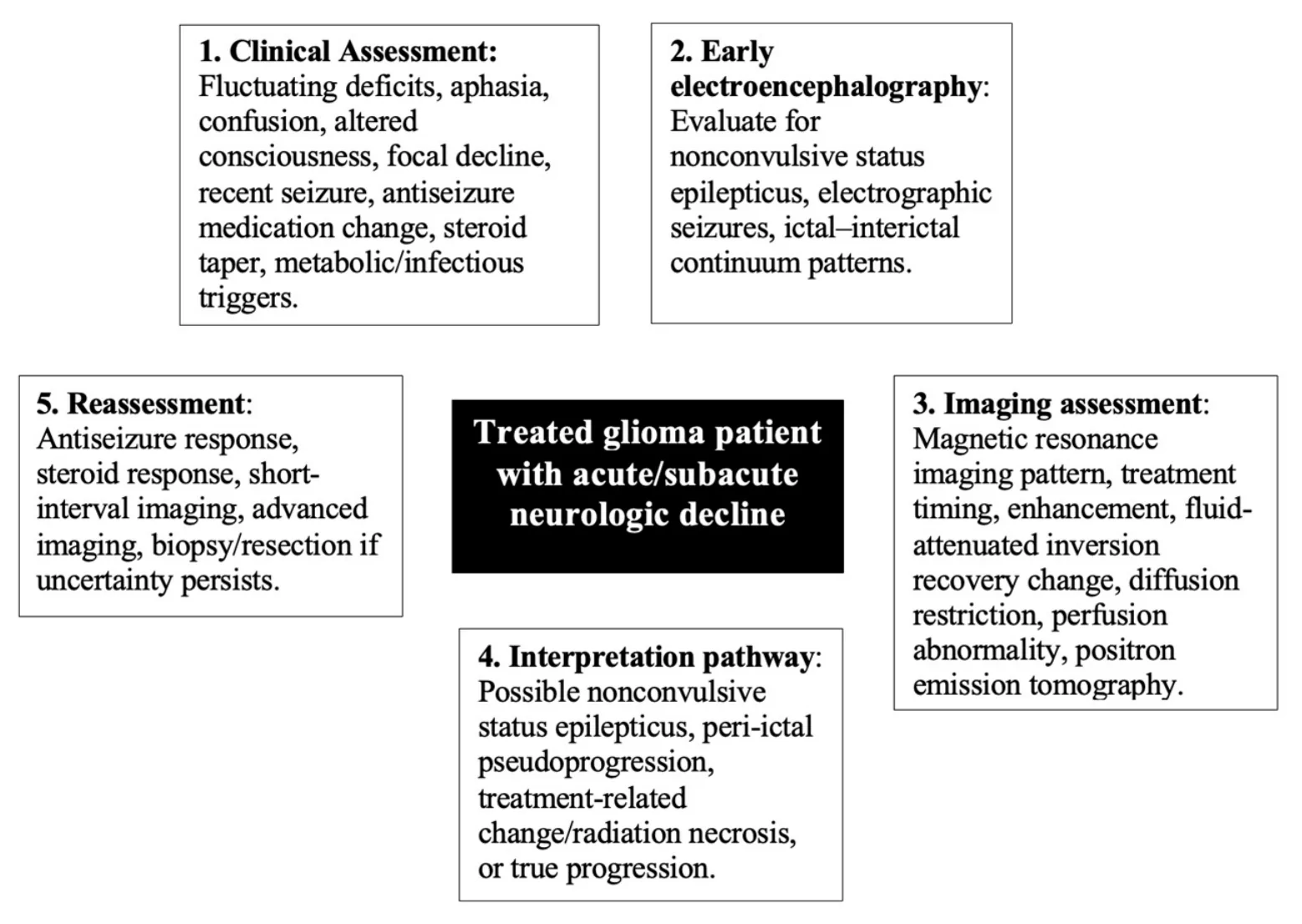
Diagnostic Framework: Integrated electroclinical-radiographic approach to acute neurologic deterioration in treated glioma [5,6,7,8,9,10,13,14,16,19].

EEG provides the most direct evidence for NCSE or electrographic seizures and should be considered early when neurologic decline is unexplained or fluctuating [6,10,11]. Imaging should be interpreted in relation to treatment timing and seizure timing, because peri-ictal enhancement, diffusion restriction, swelling, or hyperperfusion may overlap with treatment-related change or progression [7,8,13]. Reassessment is often decisive, as seizure-related abnormalities may improve after seizure control, treatment-related changes may stabilize or fluctuate, and true progression more often shows continued worsening [7,13,16,19]. In clinically stable patients with uncertain findings, repeat MRI within approximately 4–8 weeks provides a practical reassessment interval, with earlier imaging when neurologic deterioration persists or worsens [19,31]. Key primary studies relevant to seizure-related deterioration and diagnostic differentiation are summarized in Table 1; the major distinguishing features of NCSE, peri-ictal pseudoprogression, classic pseudoprogression, radiation necrosis, and true progression are summarized in Table 2; and Figure 1 presents the proposed clinical workflow [6,7,8,9,10,13,14,16,19,28,29,30,31,33,34].

**Table 1:** Key Primary Studies Informing the Narrative Review: Study Design, Population, Methods, Findings, and Limitations [6,7,10,28,29,30,31,33,34].

Study /Design	Population/Treatment	EEG	Imaging	Key Findings	Limitations
**Marcuse et al. [6], retrospective single-centre study**	1101 brain-tumor patients; 259 underwent EEG; 24 had NCSE	EEG-confirmed NCSE	MRI	NCSE resolved in 22/24; 18 improved clinically	Mixed tumors; EEG-selected; retrospective
**Rheims et al. [7], multicentre case series**	10 brain-tumor patients; nine long-term radiotherapy survivors	EEG when available	Serial contrast MRI	Seizure-related enhancement mimicked progression and later resolved	Small heterogeneous series; no controls
**Kaneoka et al. [10], retrospective single-centre cohort**	108 glioma patients; five had NCSE	Modified Salzburg criteria	MRI	NCSE occurred in 4.6%; all episodes were controlled, but function declined	Five cases; single-centre
**Giovannini et al. [28], observational cohort**	Adults with glioma and tumor-associated SE	Clinical and EEG assessment	Tumor imaging	Described presentation and outcomes of tumor-associated SE	Limited molecular and subtype data
**Stritzelberger et al. [29], retrospective single-centre cohort**	520 patients with de novo glioblastoma; 48 developed SE	Clinical and EEG assessment	Tumor and postoperative imaging	Nearly 10% developed SE; almost 80% were successfully treated	Single-centre; glioblastoma only
**Rickel et al. [30], retrospective multicentre cohort**	208 patients with primary or metastatic brain tumors and SE	Clinical and EEG classification	Tumor imaging	SE was associated with substantial functional decline	Heterogeneous tumors; not glioma-specific
**Nair et al. [31], case report**	Treated recurrent oligodendroglioma; seizures five years later	Not central	MRI, perfusion, spectroscopy, and FET-PET	Recurrence-like findings regressed at eight weeks without antitumor treatment	Single case; no pathological confirmation
**Taal et al. [33], retrospective cohort**	85 malignant glioma patients after radiotherapy and temozolomide	Not seizure-focused	Serial MRI	Up to half with early apparent progression had pseudoprogression	Pre-molecular; not seizure-specific
**Brandes et al. [34], glioblastoma cohort**	103 newly diagnosed patients after chemoradiotherapy	Not seizure-focused	Serial MRI and MGMT analysis	MGMT methylation was associated with pseudoprogression and outcome	Not independently diagnostic; not seizure-specific

**Table 2:** Summary of Distinguishing Characteristics: Diagnostic features of seizure-related deterioration, treatment-related change, and true progression in treated glioma [6,7,8,10,13,14,16,19].

Feature	NCSE	Peri-ictal Pseudoprogression	Classic Pseudoprogression	Radiation Necrosis	True Progression
**Typical clinical pattern**	Fluctuating aphasia, confusion, reduced consciousness, or focal deficits without convulsions [6,10,11]	Recent seizure with transient or worsening symptoms [7,8,31]	Often stable or mildly symptomatic; variable steroid response [13,19,33,34]	Subacute decline, edema, mass effect, possible steroid response [13,14]	Progressive deficits, steroid dependence, functional decline [14,16,19]
**Key diagnostic evidence**	EEG-confirmed seizure activity matching symptoms [6,10,11,12]	Seizure timing plus improvement after seizure control [7,8,31]	Treatment timing plus later stabilization or regression [13,19,33,34]	Delayed post-radiotherapy onset with convergent findings [13,14]	Persistent progression despite correcting reversible causes [14,16,19]
**MRI pattern**	Normal or peri-ictal cortical/subcortical changes [6,8,10]	Cortical/gyriform enhancement, FLAIR change, diffusion restriction, reduced ADC, or perfusion change [7,8,31]	Enhancement or edema that later stabilizes or regresses [13,19,33,34]	Necrotic enhancement, edema, mass effect, often within radiation field [13,14]	Enlarging enhancement, expanding nonenhancing disease, increasing mass effect [14,16,19]
**Temporal clues**	Abrupt or fluctuating decline at any stage [6,10]	Close relationship to seizure activity [7,8,31]	Usually early after chemoradiotherapy [13,19,33,34]	Usually months to years after radiotherapy [13,14]	Persistent worsening on serial assessment [16,19]
**Treatment response**	Clinical and EEG improvement with antiseizure treatment [6,10]	Clinical or imaging improvement after seizure control [7,8,31]	Stabilization or regression without progression-directed treatment [13,19,33,34]	May improve with steroids or bevacizumab but can persist [13,14]	Persists or worsens despite seizure control [14,16,19]
**Evidence limitations**	Mostly retrospective; limited glioma-specific cohorts [6,10,27,28,29,30]	Mainly small case series and case reports [7,31]	Broader observational evidence; not seizure-specific [13,19,33,34]	Heterogeneous evidence; tumor and treatment effect may coexist [13,14]	Usually requires convergent evidence; pathology may be mixed [13,14,16,19]
**Relative diagnostic weight**	Highest when EEG matches the clinical presentation [6,10,11,12]	Highest when seizure timing and improvement are concordant [7,8,31]	Supported by timing and later stabilization or regression [13,19,33,34]	Supported by delayed timing and convergent findings [13,14]	Highest with sustained worsening despite correction of reversible causes [14,16,19]

## 4. Discussion

### 4.1. Consequences of Misclassification and Clinical Actionability

Misclassification may have both immediate and long-term consequences for patient care and outcomes [6,7,10,13,16,19]. If NCSE is missed, a potentially reversible neurologic emergency may be mislabeled as tumor progression or irreversible decline [6,10]. This may delay antiseizure treatment, worsen functional status, and distort prognostic discussions [6,10]. If peri-ictal pseudoprogression is misdiagnosed as recurrence, patients may undergo unnecessary treatment escalation, bevacizumab exposure, biopsy, open surgery, discontinuation of effective therapy, or removal from clinical trials [7,31]. If true progression is incorrectly attributed to seizure-related change, salvage therapy may be delayed [16,19]. The evidence therefore supports neither reflexive oncologic escalation nor reflexive reassurance after seizures. Instead, seizure-related etiologies should be actively tested and incorporated into the same diagnostic workflow as treatment effect and progression [7,8,13,16,19].

In practice, assessment should proceed from stabilization and exclusion of reversible metabolic, infectious, vascular, and medication-related causes to targeted seizure history, medication review, EEG, and interpretation of MRI in relation to glioma subtype, treatment timing, and seizure timing. Routine EEG may be used initially in clinically stable patients, while continuous EEG should be considered when impaired consciousness or fluctuating deficits persist or clinical suspicion remains high. Advanced imaging may be used when conventional assessment remains equivocal, while response to antiseizure medication or corticosteroids should be regarded as supportive rather than independently diagnostic [6,7,8,9,10,13,14,15,16].

### 4.2. Remaining Gaps and Future Directions

Several limitations of this review should be acknowledged. Because this was a narrative review rather than a systematic review or meta-analysis, study selection and synthesis were thematic and may not capture every relevant report. The evidence base for peri-ictal pseudoprogression in glioma remains limited by small series and case reports, and many diagnostic features overlap across seizure-related change, treatment effect, and true progression. Accordingly, the proposed framework should be interpreted as a clinically oriented synthesis rather than a validated diagnostic algorithm.

The principal evidence gap is not whether these diagnoses are considered together in routine practice, but whether any structured combination of clinical, EEG, imaging, treatment-context, and longitudinal features improves diagnostic accuracy, management decisions, or patient outcomes. Available studies do not quantify delays in EEG acquisition, underuse of continuous EEG, inappropriate antiseizure treatment, or diagnostic misclassification specifically attributable to a lack of clinical integration. Existing glioma epilepsy reviews focus primarily on seizure mechanisms and antiseizure management [5,9], NCSE studies emphasize electrographic diagnosis and treatment [6,10,11], pseudoprogression reviews focus on imaging differentiation from recurrence [13], and RANO papers address standardized response assessment [16,19]. Peri-ictal pseudoprogression reports identify a clinically important mimic, but the evidence remains limited by small sample sizes and case-based literature [7,31]. Prospective studies are therefore needed to define how EEG, MRI, PET, clinical features, treatment timing, and longitudinal follow-up should be combined in a clinically applicable framework when a treated glioma patient deteriorates [6,7,10,13,14,16,19].

Future research should address this gap prospectively and also respond to the documented underrepresentation of seizure outcomes in glioma trials by incorporating seizure-specific endpoints, rather than relying only on retrospective clinical notes [25]. Studies should enroll treated glioma patients with acute or subacute neurologic decline and collect standardized seizure history, RANO-TREAT or similar seizure metrics, EEG or continuous EEG data, conventional MRI, advanced MRI, medication and corticosteroid exposure, treatment timing, and longitudinal outcomes [23,25]. Diagnostic endpoints should include imaging resolution, confirmed progression, functional recovery, antiseizure response, treatment change, and survival outcomes [23,25]. Such studies would allow for the development of practical diagnostic algorithms and could clarify which features most strongly predict NCSE, peri-ictal pseudoprogression, treatment effect, or true progression [6,7,10,13,16,19]. Until such evidence is available, the best-supported approach incorporates early EEG for unexplained deterioration, contextual interpretation of imaging, attention to treatment timing, and reassessment when diagnosis remains uncertain [6,7,8,10,13,14,16,19].

## 5. Conclusions

Acute neurologic deterioration in treated glioma patients requires consideration of NCSE, peri-ictal pseudoprogression, treatment-related change, radiation necrosis, true progression, and other reversible causes. Nonconvulsive status epilepticus and peri-ictal pseudoprogression are particularly important because they may produce reversible neurologic decline and imaging abnormalities that resemble progression or radiation-related injury [6,7,10,13]. This overlap supports a low threshold for EEG when deterioration is unexplained, fluctuating, or disproportionate to imaging findings, and it requires imaging interpretation in relation to seizure timing, treatment interval, corticosteroid exposure, antiseizure response, and longitudinal imaging evolution [7,8,11,13]. Although RANO criteria and advanced imaging improve response assessment, they do not fully address seizure-driven clinical and imaging confounding [14,15,16,19]. Future prospective studies should evaluate integrated diagnostic pathways combining EEG, conventional and advanced imaging, seizure history, treatment timing, therapeutic response, and longitudinal outcomes. Prospective studies are required to determine whether the proposed framework improves diagnostic accuracy, management decisions, or patient outcomes.

## Data Availability

No new data were created or analyzed in this study. Data sharing is not applicable to this article.
